# GEOGRAPHY AND LIVING ARRANGEMENT AS LONELINESS FACTORS FOR OAA NUTRITION CLIENTS DURING COVID-19

**DOI:** 10.1093/geroni/igae098.2539

**Published:** 2024-12-31

**Authors:** Heather Menne, Jason Osborne, Claire Pendergrast

**Affiliations:** Miami University, Oxford, Ohio, United States; Miami University, Oxford, Ohio, United States; Syracuse University, Delmar, New York, United States

## Abstract

Loneliness is increasingly understood as a public health crisis. Multiple risk factors for increased loneliness in older adults have been identified in previous studies, including age, gender, race, geographic location, and living alone. Social distancing efforts during the COVID-19 pandemic may have increased loneliness among older adults. This study uses two cross-sectional waves of the National Survey of Older Americans Act Participants (NSOAAP) from 2019 and 2021 to expand understanding and identify possible points of intervention to increase social support for vulnerable older adults. Results reveal that while home-delivered meal participants have higher overall levels of loneliness than congregate meal participants. Those receiving home-delivered meals did not experience changes in loneliness during the COVID-19 pandemic, and did not differ significantly by age, geographic location, or living arrangement. Congregate meal participants’ loneliness was lower before the pandemic but increased during the first year of the pandemic, particularly for participants aged 65-74, those living in suburban or rural areas, and those living alone. The significant differences based on geography and living arrangement for congregate meal clients (but not home-delivered meal clients) suggest the need for further investigation as to whether living arrangement has a buffering effect on loneliness or whether the closing and less access to congregate meals sites impacted loneliness. These findings suggest opportunities for policymakers and aging services providers who seek to increase social engagement among older adults who participate in Older Americans Act (OAA) nutrition programs.

